# Acupuncture for Parkinson's Disease: Efficacy Evaluation and Mechanisms in the Dopaminergic Neural Circuit

**DOI:** 10.1155/2021/9926445

**Published:** 2021-06-15

**Authors:** Yadan Zhao, Zichen Zhang, Siru Qin, Wen Fan, Wei Li, Jingyi Liu, Songtao Wang, Zhifang Xu, Meidan Zhao

**Affiliations:** ^1^Research Center of Experimental Acupuncture Science, Tianjin University of Traditional Chinese Medicine, Tianjin 301617, China; ^2^Suzuka University of Medical Science, Suzuka 5100293, Japan; ^3^School of Acupuncture & Moxibustion and Tuina, Tianjin University of Traditional Chinese Medicine, Tianjin 301617, China

## Abstract

Parkinson's disease (PD) is a chronic and progressive neurodegenerative disease caused by degeneration of dopaminergic neurons in the substantia nigra. Existing pharmaceutical treatments offer alleviation of symptoms but cannot delay disease progression and are often associated with significant side effects. Clinical studies have demonstrated that acupuncture may be beneficial for PD treatment, particularly in terms of ameliorating PD symptoms when combined with anti-PD medication, reducing the required dose of medication and associated side effects. During early stages of PD, acupuncture may even be used to replace medication. It has also been found that acupuncture can protect dopaminergic neurons from degeneration via antioxidative stress, anti-inflammatory, and antiapoptotic pathways as well as modulating the neurotransmitter balance in the basal ganglia circuit. Here, we review current studies and reflect on the potential of acupuncture as a novel and effective treatment strategy for PD. We found that particularly during the early stages, acupuncture may reduce neurodegeneration of dopaminergic neurons and regulate the balance of the dopaminergic circuit, thus delaying the progression of the disease. The benefits of acupuncture will need to be further verified through basic and clinical studies.

## 1. Introduction

Parkinson's disease (PD) is a chronic and progressive neurodegenerative disease typically affecting middle-aged and elderly people. The key clinical manifestation is the dysfunction of voluntary motor regulation, including bradykinesia, resting tremor, myotonia, and postural instability [1]. In addition to motor dysfunction, nonmotor symptoms may arise during progression of PD, including cognitive dysfunction, emotional disorders such as apathy, depression, anxiety, and hallucinations, and autonomic nervous dysfunction, such as constipation, hyposmia, and sleep disorders [2]. PD has a prevalence of 0.3% and is thus the second most common neurodegenerative disease after Alzheimer's disease [3]. Tragically, the physical status of PD patients declines even when treated, often deteriorating to severe disability and death. Treatment costs impose enormous financial demands on patients and their families, incurring over US$14 billion annually [4].

Pathologically, PD is characterized by the loss and degeneration of dopaminergic neurons in the substantia nigra (SN), *α*-synuclein (*α*-syn) deposition, and Lewy body formation [5]. Current treatment options for PD include medication, surgery, and exercise therapy, although medication is the first choice for PD, with the most commonly prescribed drugs being dopaminergic and anticholinergic agents [2]. These have been shown to control the initial symptoms of patients rapidly and effectively [6], but after a so-called “honeymoon period” of treatment, the effectiveness often decreases and medication may no longer be beneficial for patients with advanced PD. The fundamental reason for this phenomenon is that existing anti-PD drugs offer only symptomatic treatment but cannot delay disease progression. Moreover, anti-PD drugs can also lead to considerable side effects which can aggravate PD symptoms, and some of which still lack effective treatment [7]. Many patients with PD have therefore turned to complementary and alternative therapies which may be used in the early stages of PD or prior to the use of levodopa (L-DOPA) to relieve symptoms and delay PD progression, with the potential expectation that these therapies may also reverse or delay neuronal degeneration [8].

Acupuncture is an economical therapy in traditional Chinese medicine originating from China where it has been developed for more than 4,000 years. It can promote health or treat diseases via various different techniques such as manual acupuncture (MA), electroacupuncture (EA), moxibustion, and acupressure on specific anatomical locations (i.e., acupoints) [9]. Numerous clinical trials have shown that acupuncture exhibits significant therapeutic benefits for PD patients, reducing both motor and nonmotor symptoms. Moreover, acupuncture may help to reduce the dose and frequency of anti-PD drugs as well as alleviate their side effects [10]. The therapeutic mechanisms of acupuncture have also been shown to involve a reduction of mitochondrial dysfunction, oxidative stress, protein aggregation, impaired autophagy, and neuroinflammation [11]. In the present review, we aim to address the clinical efficacy and potential mechanisms of acupuncture in PD treatment, providing novel insights for the clinical application of acupuncture for the reversal the degeneration of dopaminergic neurons and delay of PD progression.

## 2. Methods

### 2.1. Search Strategy

We searched the PubMed database for studies published between January 2000 and December 2020 containing the keywords “Acupuncture” and “Parkinson's disease”. Only studies in English language were included. This search identified relevant 183 articles.

### 2.2. Study Selection

**Figure 1:**
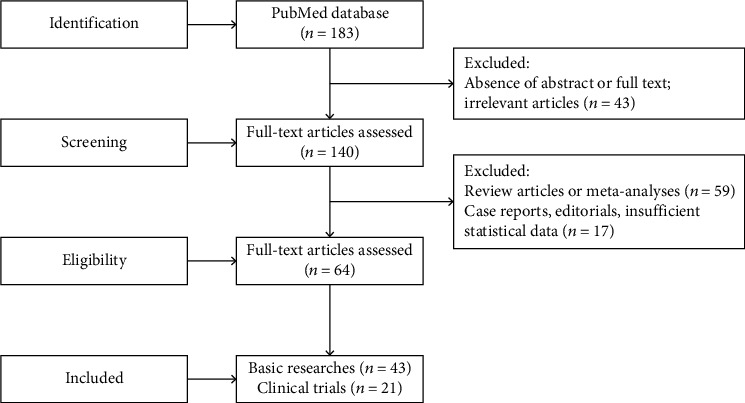
Flow chart of the review processes.

Following the search engine selection of 183 articles, a manual search was performed by screening the reference list for articles that met our inclusion criteria based on the titles and abstracts. We excluded 43 articles due to absence of an abstract, unavailability of the full text, or irrelevance to the subject, leaving 140 articles, including 43 basic research articles, 38 clinical research articles, and 59 review articles or meta-analyses. The full texts of the 43 basic research and 38 clinical research articles were obtained and assessed carefully. Out of the 38 clinical studies, 17 were excluded as they described single case reports, editorials, and uncompleted or uncontrolled trials, resulting in 21 clinical articles meeting our inclusion criteria. All 43 basic research articles met our inclusion criteria. Thus, a total of 64 articles were included in our review. A flow chart of this search process is illustrated in Figure 1.

### 2.3. Data Extraction

Two authors independently assessed the titles and abstracts of the retrieved articles and evaluated the full-text articles. The data from finally included articles were validated and extracted according to the predefined criteria. Any disagreement was resolved by discussion between the authors.

## 3. Clinical Efficacy of Acupuncture in Parkinson's Disease Patients

Acupuncture is commonly and globally used as both an adjuvant therapy and monotherapy for PD treatment [8]. Comprehensive evidence for the efficacy and safety of acupuncture in PD has been provided by a total of twelve systematic reviews (SRs) or meta-analyses (MAs) assessing this topic, involving 179 randomized controlled trials (RCTs) with a total of 11,717 participants [12]. These SRs/MAs have compared the effects of acupuncture combined with medication versus medication alone, demonstrating better efficacy at symptom alleviation with the combination of acupuncture with medication compared to medication alone [13]. The Unified Parkinson's Disease Rating Scale (UPDRS) provides a comprehensive, efficient, and flexible means of monitoring PD-related disabilities and impairments as well as treatment evaluation, assessing behavior and mood, activities of daily living, motor functions, and therapeutic complications [14]. Acupuncture combined with dopaminergic drugs was superior to medication only for improving the UPDRS score [15]. Interestingly, when comparing acupuncture only with medication only, there is no significant difference in the scores. Moreover, evidence-based medicine (EBM) studies have analyzed 13 parallel RCTs and found no difference in the efficacy of acupuncture compared with medication for PD treatment [10], suggesting that acupuncture may achieve an efficacy close to that of medication. The Webster Scale is applied to assess the clinical severity of PD, rating levels of akinesia, rigidity, upper limb swing, gait, tremor, facial appearance, speech, and self-care [16]. Compared with medication only, acupuncture and medication combined have been shown to result in a greater Webster score reduction, indicating an alleviation of clinical severity [12], and compared with medication only, acupuncture alone similarly exhibited a positive effect for decreasing the Webster score [10], suggesting that acupuncture alone may be able to effectively reduce the severity of PD. EBM reviews reveal that even with an optimized oral dose of L-DOPA, there is a risk for a worsening of movement disorders, and a variety of anti-PD drugs may also induce or exacerbate nonmotor symptoms [17]. Hence, treatment with L-DOPA and other anti-PD drugs should be delayed as much as possible in patients with early-stage PD. If acupuncture achieves a similar therapeutic effect to currently available medication, it may represent a new therapeutic option for PD, especially for early stages. There is no evidence for any serious adverse reactions associated with acupuncture, suggesting that it is a relatively safe choice for PD patients. Therefore, the current evidence indicates that acupuncture may enhance the therapeutic efficacy of drugs and reduce the required dose of medication, thus also alleviating adverse effects.

Besides UPDRS and Webster scales, there are more targeted evaluation criteria corresponding to different PD symptoms [18]. The characteristics of the 15 clinical studies included in our analysis are summarized in Table 1. Using wearable sensors, Lei et al. [19] found that EA had overall beneficial impact on different temporal and spatial parameters of gait (gait speed and stride length) as well as dynamic posture control (increase of midswing angular velocity and decrease of double support). Kong et al. [20] evaluated changes in the General Fatigue score of the Multidimensional Fatigue Inventory after acupuncture treatment and found that 5-week acupuncture treatment effectively alleviated moderate and severe fatigue of PD patients. These results are similar to reports of an RCT by Kluger et al. assessing the relief of PD with fatigue by acupuncture [21]. Using functional magnetic resonance imaging (fMRI), Yu et al. [22] found enhanced connectivity in four junctions via fMRI after acupuncture. There was a significant correlation between changes in functional connectivity and the King's Parkinson's Disease Pain Scale, indicating that acupuncture relieves pain in PD patients via modulation of brain regions related to both sensory-discriminative and emotional aspects. In an unblinded trial including 20 PD patients, no adverse reactions were reported with an improvement of individual symptoms including tremor, walking, writing, bradykinesia, pain, sleep, depression, and anxiety in 85% of patients [23]. An RCT by Cristian et al. [24] showed a trend toward an improvement in the Parkinson's Disease Questionnaire, daily living activities, and UPDRS motor score following acupuncture, suggesting that acupuncture has a potential effect for alleviation of both movement and nonmotor symptoms including pain, depression, and autonomic symptoms. Using the Parkinson Disease Sleep Scale, Aroxa et al. [25] found that acupuncture was beneficial for the alleviation of sleep disorders in PD patients. Furthermore, acupuncture may be beneficial for improving oral cavity function as observed by increased mean tongue pressure and decreased average saliva swallowing reflex latency in PD patients after acupuncture [26].

Taken together, the clinical studies above have concluded that acupuncture exhibits a comprehensive beneficial effect on symptoms associated with dyskinesia, psychology, behavior, emotion, and cognition of PD patients. Additionally, there are various types of acupuncture methods, such as MA, EA, and bee venom acupuncture (BVA). A combination of body and scalp acupoints is typically used in PD patients, with scalp acupoints located in the occipital, frontal, and parietal lobes as well as in motor and chorea-tremor controlled areas [33]. It has been shown that scalp acupoints cannot only directly activate and adjust functional areas of the corresponding cerebral cortex but also convey certain therapeutic advantages for movement disorders [34]: EA at scalp acupoints has been found to reduce the loss of dopaminergic neurons in the SN and delay the degeneration of dopaminergic neurons, thereby alleviating clinical symptoms and postponing the progression of PD [35]. Therefore, scalp acupoints are an indispensable part of PD acupuncture treatment. In clinical PD acupuncture trials, the acupoints LR3 (*Taichong*), GB34 (*Yanglingquan*), and ST36 (*Zusanli*) were the primary body acupoints used, followed by LI4 (*Hegu*), LI11 (*Quchi*), and SI3 (*Houxi*) [2]. Previous reports showed that GB34 significantly improves PD symptoms, in particular motor dysfunction, and attenuates dopaminergic neuronal loss in animal models of PD [36]. Other acupoints such as ST36 [37] and LR3 [38] displayed the same effects. Several preclinical randomized studies have demonstrated that acupuncture stimulation at GB34, GV20, and LR3 modulates PD-associated brain regions and results in an amelioration of locomotor function closely associated with a reduction of neuronal apoptosis in the striatum (ST) and SN [39]. Thereby, these three acupoints have been recommended as the basic setting of acupuncture in clinical treatment of PD according to the World Health Organization standards [40].

In the clinical application of acupuncture, acupoints are also selected according to different PD symptoms and complications [26, 27]. For instance, acupoints LR3, KI6 (*Zhaohai*), and KI7 (*Fuliu*) located in the lower leg are expected to relieve pain; acupoints HT3 (*Shaohai*), LI7 (*Wenliu*), and SI3 are stimulated in severe tremor; moxibustion at acupoints SP21 (*Dabao*) and LR14 (*Qimen*) or acupuncture at acupoints DU16 (*Fengfu*), DU14 (*Dazhui*), and Extra (*Baliao*) is used for obvious neck stiffness; acupoints DU4 (*Mingmen*) and UB23 (*Shenshu*) are stimulated for low back pain; BL15 (*Xinshu*), LR3, and SP6 (*Sanyinjiao*) points for depression and anxiety; acupoints CV23 (*Lianquan*) and ST4 (*Dicang*) for dysphagia; acupoints SP15 (*Daheng*), ST25 (*Tianshu*), CV6 (*Qihai*), and SJ6 (*Zhigou*) for constipation; acupoints CV11 (*Jianli*) and P6 (*Neiguan*) for fullness in the chest and epigastrium; and LR3, KI7, and BL18 (*Ganshu*), BL15, GB34, and UB13 (*Feishu*), UB20 (*Pishu*), and CV6 for hot flashes and paroxysmal sweating.

Taken together, a host of clinical studies have shown that acupuncture is beneficial for PD patients, especially for reducing the required dose of medication and related side effects [41]. However, there are several limitations in current acupuncture studies. First, outcome measures of most studies are subjective and vulnerable to potential biases, potentially reducing the credibility of the efficacy of acupuncture. Second, only few articles have defined acupoints according to different pathological stages or symptoms. Appropriate acupoints should be added according to different symptoms of patients. Third, the acupuncture protocol and stimulating parameters differ between trials. In the future, research on acupuncture treatment for PD should be carried out with higher methodological quality, optimized acupoint selection, frequency, duration, and study of long-term effects.

## 4. Mechanism of Acupuncture Protecting from Dopaminergic Neurodegeneration

Extensive studies have shown that genetic predisposition [42], environmental factors [43], and a variety of intracellular and extracellular pathogenic factors contribute to the development of PD, which is characterized by selective loss of dopaminergic neurons in the SN, depletion of dopamine (DA) in the ST, and the formation of Lewy bodies that are mainly composed of *α*-syn [44]. The reduction of striatal DA levels disrupts the neurotransmitter balance in the basal ganglia and thalamus, resulting in motor symptoms such as paralysis agitans, bradykinesia, and rigidity [45]. Loss of DA and changes in neurotransmitters such as serotonin (5-HT), norepinephrine (NE), *γ*-aminobutyric acid (GABA), and glutamate (Glu) in different brain regions and the peripheral nervous system lead to the occurrence of nonmotor symptoms, such as autonomic nervous dysfunction, neuropsychiatric disorders, sleep disorders, and gastrointestinal symptoms [46].

After reviewing 31 basic studies on acupuncture treatment of PD models (Table 2), we found that MA and EA were most commonly utilized, although BVA, moxibustion, acupoint injection, and laser acupuncture were also reported. It is worth mentioning that EA at high frequencies shows better efficacy than low frequencies in some animal models of PD [47, 48]. The most commonly selected acupoint was GB34 followed by LR3. Thirty studies showed that acupuncture improves motor function in PD models as assessed by the rotarod test, the cylinder test, and the pole test. Basic studies suggest that acupuncture may achieve its effectiveness for PD treatment by preventing the DA neurons from *α*-syn aggregation, apoptosis, oxidative stress, and modulating neuroinflammation and the basal ganglia circuit around DA neurons, which we have outlined in detail below.

### 4.1. *α*-Synuclein Aggregation

During the pathogenesis of PD, soluble *α*-syn monomers are thought to progressively aggregate to large insoluble *α*-syn fibrils, called Lewy bodies [79]. Overproduction and aggregation of *α*-syn furthermore induces mitochondrial dysfunction, oxidative stress, and neuroinflammation, leading to damage of dopaminergic neurons in the SN and ST.

It is suggested that acupuncture may inhibit increased levels of *α*-syn for its neuroprotective effects. Serum/glucocorticoid-regulated kinase 1 (SGK1) is a serine threonine-specific protein kinase which may regulate *α*-syn. Yeo and Lim [56] found that acupuncture at GB34 and LR3 upregulated SGK1 and inhibited an *α*-syn increase. In SGK1 siRNA knockdown SH-SY5Y cells, the authors observed a downregulation of SGK1 in dopaminergic neurons along with an increase in *α*-syn expression, suggesting that acupuncture may inhibit the increase of *α*-syn expression by downregulation of SGK1. Other research shows that overexpression of SGK1 exerts neuroprotective functions by reducing the production of reactive oxygen species (ROS) and mitochondrial dysfunction [80]. It has been reported that in the process of *α*-syn aggregation, phosphorylation at serine 129 increases the neurotoxicity of *α*-syn. A previous study has shown that acupuncture suppressed *α*-syn levels in the SN and ST and inhibits increased levels of p-*α*-syn 32 and p-*α*-syn 16 at serine 129 in the nigral dopaminergic neurons, providing evidence that acupuncture may reduce neurotoxicity via inhibiting the level of *α*-syn and p-*α*-syn, thereby protecting dopaminergic neurons [57].

Acupuncture has also been demonstrated to promote the degradation of *α*-syn by restoring autophagy. Autophagy is essential for the removal of toxic *α*-syn aggregates in order to maintain intracellular homeostasis [81]. Mammalian target of rapamycin (mTOR) is a negative regulator of cellular autophagy and it has been shown that 1-methyl-4-phenyl-1,2,3,6-tetrahydropyridine (MPTP) upregulates microtubule-associated protein 1 light chain 3 II (LC3-II) in a PD model, while downregulation lysosomal-associated membrane protein 1 (LAMP1, lysosomal structural protein) indicates that an impairment of lysosomes and the interruption of autophagosome-lysosome fusion may lead to an accumulation of autophagosomes in the substantia nigra pars compacta (SNpc) of MPTP mice [58]. Tian et al. observed that after acupuncture treatment for 4 days, LC3-II was reduced by approximately 40% and LAMP1 by approximately 20%, and more than 50% of *α*-syn in the SNpc was cleared, suggesting that acupuncture at GB34 restores lysosomal structures and reduces the accumulation of autophagosomes, enhancing the clearance of autophagosomes and degradation of *α*-syn. This group further found that acupuncture did not change levels of upstream proteins of lysosomal autophagy system (LAS), p-mTOR, p-p70s6k, and ULK1, which indicated that acupuncture activates the independent mTOR pathway to enhance the autophagic clearance of *α*-syn. After activation of mTOR, p70S6K and 4E-binding protein 1 (4E-BP1) are activated to form p-p70S6K and p-4E-BP1, which then inhibit autophagy. Wang et al. [59] found that moxibustion at ST36, CV4 (*Guanyuan*), and GV16 (*Fengfu*) decreased p-mTOR, p-p70s6k and *α*-syn levels while increasing LC3-II levels, suggesting moxibustion exerts neuroprotective effects by promoting clearance of *α*-syn and enhancing autophagy via the mTOR pathway. As rapamycin (mTOR antagonist) treatment results in considerable side effects in PD patients such as dyslipidemia, antiproliferative toxicity, and renal dysfunction [82], acupuncture may represent as the alternative strategy to target mTOR.

### 4.2. Apoptosis

It is established that nigral dopaminergic neurons in PD patients undergo apoptosis and the formation of apoptotic bodies [83]. Acupuncture may possess antiapoptosis properties via blocking apoptosis pathways of dopaminergic neurons. BVA has been found to decrease caspase-3 activity and downregulate caspase-3 and Bax gene expression, suggesting that it may inhibit the mitochondrion-dependent apoptotic pathway of dopaminergic neurons [84]. MA and acupoint injection exhibited similar effects: MA or acupoint injection at ST36 has been shown to downregulate Bax, cytochrome C, and upregulate Bcl-2 [65]. Park et al. [61] found that p53 may be involved in the neuroprotective effect of acupuncture: 40 of 76 differentially expressed genes following acupuncture were involved in the p53 signaling network, and conditionally knocking down p53 pathway genes in midbrain dopaminergic neurons attenuated the neuroprotective effect of acupuncture. The c-Jun N-terminal kinase (JNK) signaling may mediate apoptosis through phosphorylation of c-Jun [85]. Doo et al. [62] observed that BVA at GB34 can rescue dopaminergic neurons from apoptosis by inhibiting c-Jun activation in MPTP mouse, while another study suggested that MA at GB34 does not change p-c-Jun levels [69]. Therefore, the effects of different acupuncture stimulation methods on JNK need to be further clarified.

Several reports have established neurotrophic factors (NTFs) as a major player in the neuroprotective effects of acupuncture in PD treatment. Liang et al. [63] found that long-term high-frequency EA at GV14 and GV20 effectively prevented the degeneration of ventral dopaminergic neurons and upregulated the levels of brain-derived neurotrophic factor (BDNF) in the ventral subregions of the midbrain, which induced the regeneration of injured dopaminergic neurons. Subsequently, the authors also reported that EA at 2 Hz increased glial cell-derived neurotrophic factor (GDNF) mRNA in both sides of the globus pallidus, and 100 Hz EA increased GDNF mRNA in both sides of the globus pallidus and unlesioned side of SN pars reticulata, speculating that EA could regulate the retrograde transport of GDNF from ganglion to SN and restore the balance of different nuclei in the basal ganglia circuit which contributes to the behavioral improvement of medial forebrain bundle- (MFB-) lesioned rats [48]. Another study confirmed that EA upregulated BDNF and GDNF mRNA in the SN of PD models [64]. Tyrosine kinase receptor B (TrkB) is a high-affinity BDNF receptor whose activation results in the maintenance of neuronal differentiation and survival [86]. Acupuncture at GB34 and LR3 increases the TrkB expression in the damaged SN of 6-hydroxydopamine- (6-OHDA-) induced PD rats [66]. TrkB consists of different subtypes, including full-length (TrkB FL) and truncated (TrkB T1 or T2) TrkB. TrkB T1 is regarded as a dominant negative form of TrkB, which may suppress the neurotrophic activity of the BDNF/TrkB signaling pathway [87]. Hence, balancing TrkB FL and TrkB T1 is essential for neuroprotection [88]. It has been reported that the TrkB inhibitor (K252A) eliminates the neuroprotective effect of EA, and another study revealed that EA may reverse the imbalance between TrkB FL and TrkB T1 to upregulate p-Akt, p-ERK1/2, and BDNF [68]. 50 Hz EA at GB34 and LR3 has been shown to increase BDNF and downstream p-Akt levels [67], improving rotational behavior in a rat model of unilateral MPP injury, and another study revealed that inhibition of the PI3K/Akt signaling pathway blocked the protective effect of acupuncture on DA neurons [69]. Acupuncture at GB34 combined with KD5040 downregulated pI*κ*B*α* and upregulated pAkt, pGSK3*β*, pERK, pCREB, and BDNF, which significantly improve motor function [70]. Therefore, the results presented above suggest that BDNF/TrkB and their downstream signaling pathways may mediate the neuroprotective effects of acupuncture in PD treatment. Besides, acupuncture has been found to increase the number of 5-bromo-2′-deoxyuridine- (BrdU) positive cells and to restore neurogenesis in the subventricular zone [71], which provides a new pathway for studying the molecular mechanisms of acupuncture treatment for PD.

### 4.3. Oxidative Stress

Aggregation of free radicals causes lipid peroxidation, which leads to excessive oxidation during the process of protein synthesis in DA neurons, destroying the structure of the cellular membrane and ultimately resulting in the death of DA neurons. The neuroprotective effects of acupuncture treatment may be mediated through the regulation of antioxidant systems, as a study reported that MA at GB34, LR3, ST36, and SP10 increases levels of superoxide dismutase (SOD), glutathione (GSH), and glutathione peroxidase (GSH-Px), and decreases levels of malondialdehyde (MDA), along with improved rotarod behavior [38], and similar results were also observed in EA and BVA treatment. For example, high-frequency EA at ST36 and SP6 increases levels of GSH and SOD and decreases striatal H_2_O_2_ and MDA levels [47]. BVA at GB34 increases GSH and paraoxonase-1 activities and decreases MDA levels [84]. In addition to increased SOD and catalase (CAT) activities, Lee et al. also observed upregulated DJ-1, which exists widely in peripheral tissues, neurons, and glial cells, playing an essential role in antioxidation via regulating the activity of SOD and CAT [72]. It is therefore speculated that the elevation of DJ-1 caused by acupuncture at GB34 may exert antioxidant effects by enhancing the activity of striatal SOD and CAT. These results indicate that acupuncture protects DA neurons from oxidative stress by restoring the balance between oxides and antioxidants.

### 4.4. Neuroinflammation

Microglia, the primary immune cells of the central nervous system, play a vital role in the neuroinflammation in PD [89]. Injury signaling in degenerated DA neurons can shift microglia to a pro-inflammatory “M1” phenotype, resulting in the release of ROS and cytokines such as tumor necrosis factor-*α* (TNF-*α*), interleukin-1*β* (IL-1*β*), interleukin-6, and interleukin-12, which exacerbate oxidative stress and inflammation, ultimately leading to DA neuron apoptosis [90]. Inducible nitric oxide synthase (iNOS) expressed in glial cells causes nitric oxide production, which in turn activates microglia in conjunction with various proinflammatory “M1” phenotype cytokines. Acupuncture at GB34 and LR3 has been shown to attenuate the expression of macrophage antigen complex-1, a marker of microglial activation, and mitigate increases in cyclooxygenase-2 (COX-2) and iNOS expression in an MPTP-induced PD models [73]. Similarly, striatal DA levels were shown to increase from 46% to 78% within 7 days, suggesting that acupuncture exerts neuroprotective effects by attenuating MPTP-induced glial activation and neuroinflammation. Injecting choroid plexus cells at ST36 likewise decreased iNOS and COX-2 expression and enhanced exercise capacity of MPTP-induced mice [37], while BVA at GB34 has been suggested to protect dopaminergic neurons by downregulating inflammatory factors such as TNF-*α* and IL-1*β* [84].

Acupuncture can also inhibit neuroinflammation by regulating the brain-gut axis, thus alleviating movement dysfunction in PD. An altered gut microbiota has been reported to induce microglial activation and neuroinflammation, which may promote *α*-syn overexpression and contribute to motor dysfunction in PD models [91]. Jang et al. [65] observed that acupuncture changed the relative abundance of *Butyricimonas*, *Holdemania*, *Frisingicoccus*, *Gracilibacter*, *Phocea*, and *Aestuariispira*, which showed significant correlations with anxiety as well as motor functions. The authors proposed that acupuncture blocks inflammatory responses and apoptosis as acupuncture increased the levels of DA fibers and neurons in the SN and ST, downregulated glial fibrillary acidic protein, ionized calcium-binding adaptor molecule 1, and nuclear factor kappa B and TNF-*α*, and restored the conversion of Bax and Bcl-2 expression in the SN and ST. In addition, the PICRUSt-predicted functional analyses based on 16S rRNA taxonomic profiles showed that acupuncture restored physiological functions such as the glutathione and methane metabolism and other PD related pathways. Collectively, the authors postulated that modulation of the gut microbial dysbiosis and inhibition of neuroinflammation may serve as the mechanisms by which acupuncture mitigates dyskinesia and protects dopaminergic neurons in PD. Moreover, acupuncture can promote anti-inflammation by regulating inflammatory factors and the iron homeostasis in the intestine, so as to improve PD motor symptoms. Iron aggregation in the SN is an early feature of PD. Activated microglial cells regulate the import of DMT1 from microglia and downregulate the export of ferritin via FPN1, leading to accumulation of iron in microglia and ultimately damaging neurons [92]. Acupuncture at CV12 (*Zhongwan*), ST25, and CV4 not only reduced *α*-syn expression in the duodenum and inflammatory factors such as IL-1*β* and TNF-*α* in the serum and duodenum but also balanced ferritin import through DMT1 and ferritin export via FPN1 and ultimately reducing iron accumulation in the duodenum and SN [74]. It is worth mentioning that CV12, ST25, and CV4 are acupoints for the treatment of gastrointestinal symptoms, which are thought to inhibit neuroinflammation by influencing the intestinal canal to relieve movement disorders. Acupuncture has been shown to provide additional potential benefits for the brain-gut axis [93], highlighting the brain-gut-neuroinflammation-PD axis as a novel area for research.

## 5. Neural Circuit Alterations Underlying the Efficacy of Acupuncture Treatment for Parkinson's Disease

The measurement of neural activity provides more direct experimental evidence for the study of the mechanisms involved in the development and treatments of PD. Functional magnetic resonance imaging (fMRI), positron emission tomography (PET), and single-photon emission computed tomography (SPECT) have been used to measure alterations in neural signals caused by acupuncture [94]. The pathological hallmark of PD is the loss of dopaminergic neurons in the SNpc, which leads to insufficient DA projecting to the ST and cerebral cortex and results in abnormal regulation of the neural circuitry of the basal ganglia [95]. Therefore, the functional connectivity of the SN, caudate nucleus, and thalamus, which are all part of the basal ganglia circuit, is impaired in PD patients [96]. Dopaminergic neural pathways, including the basal nuclei, thalamus, and limbic system, are also impaired in animal models of PD [97]. Consequently, Tables 3 and 4 summarize the characteristics of 6 clinical studies and 12 preclinical studies assessing the neural circuit.

### 5.1. Evidence for Neural Circuit Regulation by Acupuncture Based on Clinical Imaging Studies

Using fMRI imaging, Chae et al. [98] found that acupuncture stimulation of GB34 activated regions associated with intensified motor function as evidenced by increased BOLD signals (the index of brain activation), illustrating that acupuncture may promote the improvement of motor functions in PD patients via the basal ganglia-thalamic-cortical circuit. Furthermore, neural responses of PD patients at resting state were lower than in healthy controls in extensive brain regions including the putamen, thalamus, and supplementary motor area. This variation is secondary to DA deficiency and is related to PD severity [110]. Yeo et al. performed acupuncture stimulation in 12 PD patients and 12 healthy volunteers and compared whole-brain fMRI images of the two groups before and after acupuncture treatment. The authors found that acupuncture at GB34 increased brain activation in regions impaired by PD, such as the SN, caudate, thalamus, and putamen [99], and moreover activated the prefrontal cortex, precentral gyrus, and putamen in PD patients [100]. These results support the hypothesis that PD rehabilitation may be related to modulation of areas associated with PD by GB34 acupuncture stimulation. Besides, the dyskinesia represented by tremor may also be closely related to the activity of the compensatory circuit, in particular the cerebello-thalamo-cortical (CTC) circuit, after the basal ganglia circuit is damaged [111]. Li et al. [101] demonstrated that acupuncture alleviated PD tremor by stimulation and modulation of the cerebellum, thalamus, and motor cortex, in connection with the CTC circuit. Meanwhile, by adjusting neural activity within cognitive brain regions which connect with the CTC circuit, acupuncture may contribute to enhancing movement and improving the daily life activities. The thalamus, originally thought to be a passive transmissive structure for sensory information, plays an indispensable role in the CTC pathway along with the cerebellum and is hypothesized to be involved in movement and balance. Thalamic dysfunction may result in dyspnea with stiffness and tremor as well as nonmotor symptoms [112]. Modulation of the tremor site in the ventral thalamus may help to explain the effectiveness of acupuncture for controlling tremor symptoms [113]. Pathological changes of the basal ganglia are the basis of tremor in PD; however, the direct cause of tremor is the abnormal regulation of the CTC circuit, and the contact points of these two circuits may be located in the motor cortex or other brain locations [114]. Hence, we speculate that the tremor-related neural network may include several brain regions such as the sensorimotor cortex, cerebellum, thalamus, and basal ganglia. Within this network, the neural circuit represented by the basal ganglia and the CTC plays different roles in tremor. The restoration of function of PD patients by acupuncture may involve activation of compensatory brain regions to increase the survival rate of dopaminergic neurons or the activity of DA receptors, and to correct the network imbalance caused by the loss of the dopaminergic neurons in SN [41]. Huang et al. conducted two RCTs using PET [103] and SPECT scans [102] to compare the efficacy of EA combined with medication versus medication alone. After 5 weeks of acupuncture, PET scans of the EA+medication group showed an increased glucose metabolism in the temporal lobes, cerebellum, and thalamus of the less-affected hemisphere and in the parietal and occipital lobes of the severe-diseased hemisphere, while these changes were not observed in the medication-only group. The study highlighted that more brain regions exhibited increased metabolism in the less-affected hemisphere than in the diseased hemisphere and the survival rate of dopaminergic neurons and DA receptor activity were increased, indicating that acupuncture may also affect the number and activity of nigral cells in the less-affected hemisphere [103]. The SPECT study highlighted that regional cerebral blood flow (rCBF) of the frontal and occipital lobes, basal ganglia, and cerebellum in the more severely affected cerebral hemispheres increased, yet DAT levels in the basal ganglia remained unchanged after acupuncture treatment in PD patients, while L-DOPA treatment alone did not affect rCBF, but increased DAT activity in the basal ganglia of the most severely affected cerebral hemispheres [102].

These neuroimaging results illustrate that acupuncture enhances local brain activity and glucose metabolism and improves rCBF, which in turn reactivates both the dysfunctional motor and nonmotor neural circuits, ameliorating symptoms and improving overall quality of life. These neuroimaging quantifications rather than subjective findings helped to further objectively evaluate the effectiveness of acupuncture in PD.

### 5.2. Experimental Animal Model Studies Investigating the Regulation of the Basal Ganglia Circuit in Parkinson's Disease by Acupuncture

The basal ganglia circuit consists of the ST (caudate and putamen), the globus pallidus (internal and external parts), the subthalamic nucleus, and the SN, which are responsible for motor control, motor learning, emotions, and executive functions [115]. Several neurotransmitters are involved in regulating the function of the basal ganglia circuit. Once DA levels are reduced, the imbalance of DA and acetylcholine (ACh) and disorders of the neurotransmitter system contribute to the development of dyskinesia in PD [116]. GABA is the most predominant inhibitory neurotransmitter in the ST and its downstream nucleus projections are regulated by DA either via dopamine D1 receptor (D1R) or dopamine D2 receptor (D2R). SPECT imaging in PD models revealed that retained acupuncture may attenuate MPTP-induced neuronal injury [55]. Besides, fMRI studies have demonstrated that acupuncture activates the caudate nucleus, putamen, M1, cingulate gyrus, and globus pallidus externa [104], and modulates the ipsilateral parietal lobe, contralateral temporal lobe, basal ganglia, pons, and cerebellum [97], which are closely linked to the basal ganglia circuit.

Acupuncture can improve the motor function in PD models by regulation of neurotransmitters and restoration of the homeostasis of the basal ganglia circuit: first, acupuncture mediates improvements of motor function by directly affecting the dopaminergic system. Kim et al. [36] found that acupuncture improves motor function and protects dopaminergic neurons via increasing DA release, which in turn enhances the availability of DA in the synaptic cleft. Moreover, the authors observed that acupuncture reduces the expression of FosB, a marker of striatal postsynaptic dopamine depletion, and the phosphorylation of DARPP-32, which can affect postsynaptic medium spiny neurons by activating the cAMP signaling pathway. These results raised the possibility that the effects of acupuncture for the amelioration of MPTP-induced dyskinesia are mediated by upregulation of neurotransmission of postsynaptic DA and rebalanced basal ganglia circuit. EA at SP6, GB34, and ST36 with 100 Hz increased the survival of nigral DA neurons and increased the expression of striatal DAT by 253.78%, as well as elevating the levels of D1R mRNA and protein by 81.88% and 62.62%, respectively, while inhibiting increases in the D2R. These results suggested that high-frequency EA may act on both presynaptic DAT and postsynaptic DA receptors, contributing to the balance of basal ganglia output to the thalamus [105].

In terms of the regulation of other neurotransmitters, Jia et al. [106] observed that 100 Hz EA at GV14 and GV20 increased the content of nigral substance P, protected damaged DA neurons from degeneration, and reversed the abnormal elevation of GAD67 mRNA in the midbrain induced by MFB axotomy. Subsequently, the authors further found that EA at high frequency reversed increases in GABA content in the midbrain and alleviated motor coordination in MFB-lesioned rats [107]. These findings suggest that the amelioration of dyskinesia in MFB-lesioned rats may occur via restoration of the homeostasis of dopaminergic transmission and inhibition of the overoutput of GABA in the basal ganglia circuit. Sun et al. [109] reported that high-frequency EA at GV14 and GV20 reduced abnormally elevated levels of Glu and ACh on the lesioned side in the ST, improved motor coordination in MFB-lesioned rats, and protected the nigral DA neurons from degeneration. Furthermore, Glu levels were significantly correlated with the survival ratios of DA neurons in SNpc and rotational behavior, suggesting that alleviation of dyskinesia by EA may involve the downregulation of Glu and ACh in the ST. Another study found that BVA at GB34 may increase the levels of DA, NE, and 5-HT, proposing that the improvement of motor function induced by BVA may be related to increased levels of DA and NE [84].

In addition, acupuncture can also reduce L-DOPA-induced dyskinesia (LID) by regulating neurotransmitters to restore the balance of the basal ganglia circuit. As a precursor of DA, L-DOPA is used to increase DA levels and enhance synaptic DA transmission. However, after approximately 10 years of treatment with L-DOPA, most patients develop side effects, including nonmotor complications such as hallucinations, insomnia, nausea [117], and motor complications including dystonia, chorea, and athetosis, known as LID [118]. Kim et al. [108] observed that L-DOPA at half the typical dose combined with acupuncture can improve motor function. Combination therapy can also restore the levels of GABA and Glu, which are elevated by high-dose L-DOPA. Moreover, FosB expression, an early indicator of LID, is also reduced by combination therapy. These results not only suggest that acupuncture may relieve LID by balancing neurochemicals in the basal ganglia but also demonstrated a possible synergistic effect of acupuncture and L-DOPA.

In a nutshell, it is widely believed that the dysfunction of the basal ganglia circuit induced by the apoptosis of dopaminergic neurons is an essential mechanism in the development and progression of PD. The basal ganglia circuit is regulated by various neurotransmitters, and if a part in the circuit is abnormal, the function of the whole circuitry may thus be abnormal. However, since the mechanism of acupuncture for the regulation of the basal ganglia circuit which mediates improvements of PD dyskinesia has not been fully clarified so far, this paper only reviewed the published research so far. Further studies on neuropathology in this field will be beneficial to further increase the objectivity of the evaluation of the efficacy of acupuncture in PD treatment.

## 6. Conclusion

**Figure 2:**
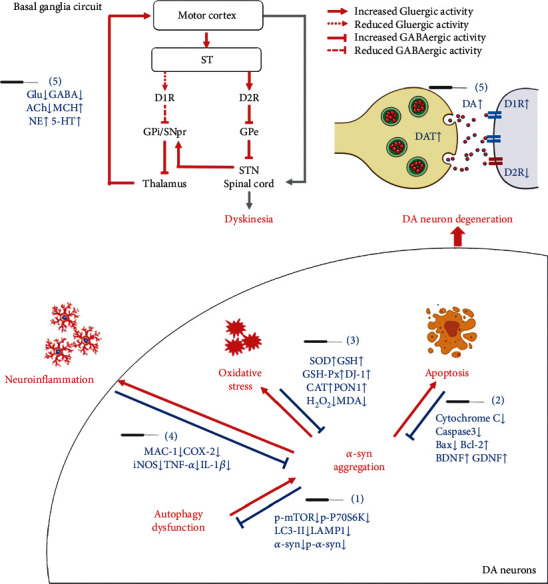
The neuroprotective mechanisms of acupuncture in the treatment of Parkinson's disease. The pathological activities associated with PD include *α*-syn aggregation, oxidative stress, apoptosis, neuroinflammation, and the disbalance of basal ganglia circuit (red). The key molecules (blue) in the above events are regulated by acupuncture to display neuroprotective effect in PD brain. ACh: acetylcholine; *α*-syn: *α*-synuclein; Bax: Bcl-2-associated X protein; Bcl-2: B cell lymphoma-2; BDNF: brain-derived neurotrophic factor; caspase-3: cysteinyl aspartate-specific proteinase-3; CAT: catalase; COX-2: cyclooxygenase-2; DA: dopamine; DAT: dopamine transporter; D1R: dopamine D1 receptor; D2R: dopamine D2 receptor; GABA: *γ*-aminobutyric acid; GDNF: glial cell-derived neurotrophic factor; Glu: glutamate; GPe: globus pallidus externus; GPi: globus pallidus internus; GSH: glutathione; GSH-Px: glutathione peroxidase; 5-HT: serotonin; IL-1*β*: interleukin-1*β*; iNOS: inducible nitric oxide synthase; LAMP1: lysosomal-associated membrane protein 1; LC3-II: microtubule-associated protein 1 light chain 3 II; MAC-1: macrophage antigen complex-1; MCH: melanin concentration hormone; MDA: malondialdehyde; NE: norepinephrine; p-*α*-syn: phosphorylated *α*-synuclein; p-mTOR: phosphorylated mammalian target of rapamycin; PON1: paraoxonase-1; p-p70S6k: phosphorylated ribosomal protein S6 kinase; SNpr: substantia nigra pars reticulate; SOD: superoxide dismutase; ST: striatum; STN: subthalamic nucleus; TNF-*α*: tumor necrosis factor-*α*.

The effectiveness of acupuncture for PD treatment has been demonstrated by several clinical, preclinical, and basic research studies. Benefits predominantly manifested via the amelioration PD symptoms and the reversal of dopaminergic neurodegeneration in the early stages of PD. The combined application of acupuncture and anti-PD drugs may enhance the therapeutic efficacy of drugs while reducing the required dose and associated side effects. Acupuncture may protect dopaminergic neurons from degeneration via antioxidative stress, anti-inflammatory, and antiapoptotic pathways, and modulating the neurotransmitter balance in the basal ganglia circuit. As shown in Figure 2, acupuncture can exert protective effects on dopaminergic neurons through multiple pathways: (1) acupuncture reduces the abnormal aggregation of *α*-syn by inhibiting the production of *α*-syn and reinstating the autophagic clearance of *α*-syn; (2) acupuncture inhibits the apoptotic pathways and upregulates the expression of NTFs to activate the downstream PI3K/AKT and MEK/ERK pathways, thus promoting the survival of dopaminergic neurons; (3) acupuncture increases the level of intracellular antioxidants to reduce the oxidative stress response after PD; (4) acupuncture inhibits the activation of microglia and the release of inflammatory factors, and reverses neuroinflammation by regulating intestinal microorganisms; (5) acupuncture regulates DA and other neurotransmitters to reinstate the balance of basal ganglia circuit and exerts a synergistic effect with L-DOPA and other anti-PD drugs. Therefore, acupuncture may represent a novel and effective strategy for the treatment of PD, particularly for halting the degeneration of DA neurons at the early stages of PD to delay PD progression, although this hypothesis needs to be further verified by high-quality basic and clinical research.

## Figures and Tables

**Table 1:** The clinical efficacy of acupuncture in the patients with Parkinson's disease.

Study	Clinical trial design	Clinical condition	Intervention	Comparison	Acupoints	Acupuncture parameters	Outcomes
Lei et al. (2016) [19]	RCT	PD patients	EA (*n* = 10)	Sham EA (*n* = 5)	GV20, GV14; bilateral foot motor, sensory area, balance area, LI4, ST36, GB34, BL40, SP6, KI3, LR3	Asymmetric biphasic square wave pulse, 100 *μ*S pulse width, “*deqi*”, 100 Hz or 4 Hz, three times a week, 3 weeks	Primary outcome: STHW↑, STFW↑, DTFW↑, and DTHW↑ Secondary outcome: UPRDS scores↓

Kong et al. (2017) [20]	RCT	PD patients with moderately severe fatigue	Acupuncture (*n* = 20)	Sham acupuncture (*n* = 20)	CV6; bilateral PC6, ST36, LI4, SP6, KI3	5-30 inches, no flicking or rotation of needles after insertion, 20 minutes, twice a week, at least 3 days apart, 5 weeks, 10 sessions	Primary outcome: MFI-GF↓ Secondary outcome: MFI-total score↓, UPDRS-motor↓

Kluger et al. (2016) [21]	RCT	PD patients with moderate or severe fatigue	EA (*n* = 47)	Sham acupuncture (*n* = 47)	GV20, GV24; bilateral LI10, HT7, ST36, SP6	0.5-1 cm, twisted three times to the right, 30 minutes, every two weeks, 6weeks	Primary outcome: MFIS (total, physical, cognitive, psychosocial)↓ Secondary outcomes: PDQ-39↓, HADS (depression)↓, ESS↓, AES↓

Yu et al. (2019) [22]	Comparison trials	PD patients with pain	Acupuncture+medication (*n* = 9)	Medication (*n* = 7)	GV20; bilateral 77.18, GB34	5-10 mm, “*deqi*” for 20 s, 30 minutes, 1-3 sessions per week, separated by at least 1 day, 8 weeks	Primary outcome: KPPS↓, UPDRS (total)↓, enhanced connectivity at the four areas, a significant correlation between functional connectivity changes and KPPS

Shulman et al. (2002) [23]	Controlled trial	Idiopathic PD patients (stages I-III)	EA (*n* = 20)	Self-control	Bilateral LI4, GB34, ST36, K3, KI7, SP6, SI3, TB5	Intensity knob: 0; electric exciter switch: 1; wave form: intermittent; twice a week, 10/16 treatments	Sleep and rest category: SIP (sleep and rest)↓

Cristian et al. (2005) [24]	RCT	PD patients (stages II-III)	EA (*n* = 7)	Sham acupuncture group (*n* = 7)	GV20; bilateral K3, K10, BL60, L3, ST41, ST36, GB34, *bafeng* points, MH6, LI4	4 Hz, 20 minutes, 5 sessions, 2 weeks	NS

Aroxa et al. (2016) [25]	RCT	Idiopathic PD patients (stages I-III)	Acupuncture+medication (*n* = 11)	Medication (*n* = 11)	Bilateral LR3, SP6, LI4, TE5, HT7, PC6, LI11, GB20	30 minutes, once a week, 8 weeks	Sleep disorders evaluation: PDSS↑

Fukuda et al. (2016) [26]	Controlled trial	PD patients (stages I-III)	Acupuncture (*n* = 13)	Self-control	Bilateral ST36, SP6, LR3, LI4, LI11, GB20, BL18, BL23	Inserted perpendicularly, 10-20 mm, 10-15 minutes, only once	Tongue function: mean tongue pressure↑, mean swallowing reflex latency↓, saliva swallow↓

Chen et al. (2015) [2]	Comparison clinical trial	PD patients	Acupuncture+medication (*n* = 20)	Medication (*n* = 20)	DU20; bilateral GB20, LI11, LI10, LI4, GB31, ST32, GB34, GB38	MA, 5-30 mm, 15 minutes, twice a week, 18 (short-term)/36 (long-term) weeks	Short-term: UPDRS (total scores and subscores I, II, III, and IV)↓, BDI-II score↓, WHO-QOL score↓ Long-term: UPDRS (total scores and subscores I, II, III, and IV)↓, BDI-II score↓

Wang et al. (2002) [27]	Controlled trial	PD patients (stages I-III)	Acupuncture (*n* = 29)	Blank control (*n* = 14)	Group 1: DU20; bilateral GB20, LI4, SP6, LR3; group 2: Extra6; bilateral LI11, SJ5, GB34, ST36, ST40, Extra21	MA at Extra6, GB13, and GB20, then electric stimulator with continuous wave for 15 minutes; the rest acupoints: uniform reinforcing-reducing method, 40 minutes; once every other day, 3 months	The Webster's cumulative scores↓, correlation analysis of ABP indices and the cumulative scores in Webster's scale↓, the latent period of V wave and the intermittent periods of III-V peak and I-V peak↓

Yeo et al. (2018) [28]	Controlled trial	Idiopathic PD patients	EA+MA (*n* = 10)	Self-control	Right GB34, LR3 (EA) Bilateral LR3, LI11, ST36, GB20, SP6, LI4, GB34 (MA)	EA: 15 minutes, 120 Hz, twice a week, 8 weeks; MA: 3 to 15 mm, 15 minutes; twice a week, 8 weeks	UPDRS (total, 1, 2, 3, and4) score↓, BDI-II scores↓, neural responses (thalamus, cingulate gyrus, anterior cingulate, lingual gyrus, parahippocampal gyrus, lateral globus pallidus, mammillary body, middle temporal gyrus, cuneus, and fusiform gyrus)↑

Cho et al. (2017) [29]	RCT	Idiopathic PD patients (stages I-IV)	Acupuncture+BVA (*n* = 29)	Sham treatment (*n* = 29)/conventional treatment (*n* = 15)	Bilateral GB20, LI11, GB34, ST36, LR3	BVA: injected diluted bee venom 0.1 ml; EA: 1-1.5 cm, rotated at 2 Hz for 10 seconds to achieve “*deqi*”, 15 minutes; twice a week, 12 weeks	Primary outcome: UPDRS (part II+III) scores↓ Secondary outcomes: UPDRS (part II and III) scores↓, gait speed↓, gait number↑, PIGD↓

Eng et al. (2006) [30]	Controlled trial	Idiopathic PD patients	Acupuncture+Yin Tui Na (*n* = 25)	Self-control	Bilateral ST42, SP3, LI11, LI15, LI20, ST7, ST36	7-10 minutes, 8-14 Hz, once a week, 6 months, 24 sessions	UPDRS (I and II) scores↓, UPDRS (III) scores↑, PDQ-39 total score↓, BDI↓

Doo et al. (2015) [31]	Controlled trial	Idiopathic PD patients	Acupuncture and BVA+conventional treatment (*n* = 11)	Self-control	Bilateral GB20, LI4, GB34, ST36, LR3	BVA: injected diluted bee venom 0.1 ml; EA: 1-1.5 cm, rotated at 2 Hz for 10 seconds to achieve “*deqi*”, 15 minutes; twice per week, 12 weeks	Primary outcome: UPDRS (part II+III) scores↓ Secondary outcomes: UPDRS (part II and III) scores↓, gait speed ↓, PDQL score↑

Ren (2008) [32]	Controlled trial	PD patients	Acupuncture+Madopar (*n* = 50)	Madopar (*n* = 30)	From TE14 to TE2, from PC2 to PC7 (areas being tapped) Bilateral TE4, LI5, PC7, SI6, LI11, LU5, PC3, HT3, TE14, LI15, SI9, LR4, KI3, ST41, SP9, GB34, BL40, GB30, BL36 (MA)	Tap: with a plum-blossom needle, until skin turns red; MA: uniform reinforcing and reducing method, 30 minutes; once a day, 10 sessions a course, 3-5 days between courses, 2 courses	Total effective rate↑, the minimum dose of Madopar needed↓

RCT: randomized controlled trial; PD: Parkinson's disease; EA: electroacupuncture; GV20: *Baihui*; GV14: *Dazhui*; LI4: *Hegu*; ST36: *Zusanli*; GB34: *Yanglingquan*; BL40: *Weizhong*; SP6: *Sanyinjiao*; KI3: *Taixi*; LR3: *Taichong*; STHW: single-task habitual walking; STFW: single-task fast walking; DTFW: dual-task fast walking; DTHW: dual-task habitual walking; UPDRS: Unified Parkinson's Disease Rating Scale; PC6: *Neiguan*; CV6: *Qihai*; MFI-GF: General Fatigue score of the Multidimensional Fatigue Inventory; GV24: *Shenting*; LI10: *Shousanli*; HT7: *Shenmen*; MFIS: Modified Fatigue Impact Scale; PDQ-39: 39-item Parkinson's Disease Questionnaire; HADS: Hospital Anxiety and Depression Scale; ESS: Epworth Sleepiness Scale; AES: Apathy Evaluation Scale; 77.18: *Shenguan*; KPPS: King's Parkinson's Disease Pain Scale; KI7: *Fuliu*; SI3: *Houxi*; TB5: *Waiguan*; SIP: Sickness Impact Profile; KI10: *Yingu*; BL60: *Kunlun*; NS: no significance; ST41: *Jiexi*; MH6: *Yinwei*; TE5: *Waiguan*; LI11: *Quchi*; GB20: *Fengchi*; PDSS: Parkinson's Disease Sleep Scale; BL18: *Ganshu*; BL23: *Shenshu*; DU20: *Baihui*; GB31: *Fengshi*; ST32: *Futu*; GB38: *Juegu*; MA: manual acupuncture; BDI-II: Beck Depression Inventory-Version 2; WHO-QOL: WHO quality of life; Extra6: *Sishencong*; SJ5: *Waiguan*; ST40: *Fenglong*; Extra21: *Huatuojiaji*; GB13: *Benshen*; ABP: auditory evoked brain stem potential; TE14: *Jianliao*; TE2: *Yemen*; PC2: *Tianquan*; PC7: *Daling*; TE4: *Yangchi*; LI5: *Yangxi*; SI6: *Yanglao*; LU5: *Chize*; PC3: *Quze*; HT3: *Shaohai*; LI15: *Jianyu*; SI9: *Jianzhen*; LR4: *Zhongfeng*; SP9: *Yinlingquan*; GB30: *Huantiao*; BL36: *Chengfu*; BVA: bee venom acupuncture; PIGD: postural instability and gait disturbance; PDQL: Parkinson's Disease Quality of Life Questionnaire; ST42: *Chongyang*; SP3: *Taibai*; LI20: *Yingxiang*; ST7: *Xiaguan*.

**Table 2:** The neuroprotective mechanisms of acupuncture in the treatment of Parkinson's disease.

Study	PD model	Intervention	Acupoints	Acupuncture parameters	Behavioral measurements	Biochemical measurements
Hong et al. (2010) [49]	MPTP mice	MA	GB34	Turned at a rate of two spins per second for 15 s	—	Grm5↓, Itpr1↓, Itpr2↓, Pde1c↓, Prkcb1↓, Plcb4↓, Atp2b3↓, Slc8a3↑, Htr5a↑, Adcy1↑, Drd5↓, Npy1r↓, Gpr156↓, Cxcl10↓, Rxfp2↑, Agtrl1↑, Inhbb↓, Cxcl5↓, IL21↓, Cox6a1↓, Uqcrq↓, Ndufc1↓, Atp5e↓, Mapt↓, Ngfr↓, Nefh↓

Choi et al. (2011) [50]	MPTP mice	MA	GB34, LR3	Turned at a rate of two spins per second for 15 s	—	TH levels in the ST and the SNpc↑, DAT↑, Ctla2a↓, EG383229↓, Ppbp↓, Ube2l6↓, EG665033↑, ENSMUSG00000055323↑, Obox6↑, Pbp2↑, Tmem150↑

Yeo et al. (2013) [51]	MPTP mice	MA	GB34, LR3	Turned at a rate of two spins per second for 15 s	—	TH in the ST, SNpc, and thalamus↑, Dusp4↑, Mafg↑, Pgm5↑, Ndph↑, Dnase1l2↑, Ucp2↓, Sp2↓, Serinc2↓

Yeo et al. (2015) [39]	MPTP mice	MA	GB34, LR3	Turned at a rate of two spins per second for 15 s	—	TH in the ST and SNpc↑, Cdh1↓, Slc6a13↓, Tph2↓, Mpzl2↓, Serping1↓, Itih2↓, Slc6a20a↓, Slc6a4↓, Ucma↓, Rdh9↓, 4921530L21Rik↑, Gm13931↑

Jeon et al. (2008) [52]	MPTP mice	EA	GB34	2/100 Hz, 1 mA for 20 minutes	Pole test: time on the pole↓	TH-positive cells in the SN↑, CyPA in the SN↑, MBP↑, Ube2N↑, LMP6↑, LMP2↑, lipocalin/cytosolic fatty acid-binding protein↑, eIF5A↑, CLP↑, ATPase, H1-transporting V1 subunit F↑, NSF↑

Wang et al. (2013) [53]	MPTP mice	EA	ST36, SP6	0/100 Hz, 1-1.25-1.5 mA for 30 minutes	Open-field test: total movement time↓, movement velocity↓, total movement distance↓	—

Kim et al. (2005) [54]	6-OHDA rats	MA	ST36	Left in acupoint for 20 minutes	Rotational behavior test: the average net number of turns/min↓	The survival rate of TH-positive neurons↑, the density of TH-positive fibers↑

Yang et al. (2011) [55]	MPTP mice	RA/EA	PC7	Left in acupoint for 15 minutes/2 and 15 Hz, alternatively, 1 mA for 15 minutes	Pole test: descending time↓	TH-positive neurons (RA)↑, DA in the SN↓, the intensity of radionuclide or radiopharmaceutical uptake (RA)↑, the peak time of [^123^I]IBZM uptake (RA)↓, HVA concentration in the SN (EA)↓

Yeo and Lim (2019) [56]	MPTP mice	MA	GB34, LR3	Turned at a rate of two spins per second for 15 s	—	TH in the ST and SNpc↑, *α*-syn in the SN↓, SGK1↑

Yeo et al. (2020) [57]	MPTP mice	MA	GB34, LR3	Turned at a rate of two spins per second for 15 s	—	TH in the ST and SNpc↑, *α*-syn in the ST and SN↓, p-*α*-syn 32↓, p-*α*-syn 16↓, p-*α*-syn 32/total *α*-syn values↑

Tian et al. (2016) [58]	MPTP mice	MA	GB34	Turned at a rate of two spins per second for 15 s, retained for 10 minutes	Rotarod test: the ORP scores↑	*α*-syn in the SNpc↓, autophagosome accumulation↓, LC3-II↓, LAMP1↓, the number and function of DA neurons↑, density of TH-positive neurons in the SNpc and ST↑, DA concentration↑, synaptophysin protein↑

Wang et al. (2018) [59]	Rotenone rats	Moxibustion	ST36, CV4, GV16	Placed 2-3 cm above each acupoint, retained for 10 minutes	Behavioral scoring [60]↓	TH-immunoreactive neurons↑, *α*-syn-positive aggregates↓, p-mTOR and p-p70S6K in the SNpc↓, LC3-II in the SNpc↑

Song et al. (2020) [37]	MPTP mice	Acupoint injection	ST36	Choroid plexus cells were injected into ST36, a depth of 3 mm, maintained for 1 week	Pole test: exercise capacity↑	TH in the SNpc↑, Bax↓, cytochrome C↓, BCL-2↑, COX-2↓, iNOS↓

Park et al. (2015) [61]	MPTP mice	MA	GB34	Turned at a rate of two spins, per second for 15 s	Rotarod test: latency time↑; cylinder test: the number of wall touches↑	TH-positive neurons in the SN↑, TH-positive fibers↑, 40 of 76 differentially expressed genes are involved in the p53 signaling network
	MPTP+P53 knockout mice	MA	GB34	Turned at a rate of two spins, per second for 15 s	—	TH-positive cells and fibers slightly↑

Doo et al. (2010) [62]	MPTP mice	BVA	GB34	—	—	TH-positive neurons in the SN↑, DA fibers in the ST↑, p-c-Jun-positive cells in the SN↓

Liang et al. (2002) [63]	MFB rats	EA	GV14, GV20	2/100 Hz, 1-2-3 mA for 30 minutes	—	TH-positive neurons on the lesion side↑, BDNF mRNA expression on the lesioned side of the VTA (100 Hz)↑, BDNF mRNA in the SNpc (100 Hz)↑

Wang et al. (2013) [64]	Rotenone rats	EA	GV16, LR3	100 Hz, 1-1.25-1.5 mA for 30 minutes	—	BDNF mRNA expression in the SN↑, GDNF mRNA expression in the SN↑

Liang et al. (2003) [48]	MFB rats	EA	GV14, GV20	2/100 Hz, 1-2-3 mA for 30 minutes	Rotational behavior test: day 14 the average net number of turns/min↓, day 28 the average net number of turns/min (100 Hz)↓	GDNF mRNA-positive cells in the SNpr (100 Hz)↑, GDNF mRNA expression in the GP of the unlesioned side (2 Hz)↑, GDNF mRNA level in both the lesioned and unlesioned side of GP (100 Hz)↑

Jang et al. (2020) [65]	MPTP mice	MA	GB34, ST36	Turned at a rate of two spins per second for 30 s	Cylinder test: the number of wall touches↑; rotarod test: latency time↑; akinesia test: latency time↓; open-field test: the total distance↑, central distance↑, the ratio of central/total distance↑, the number of crossings in the central zone↑	Dopaminergic fibers and neurons in the ST and SN↑, TH-positive neurons in the ST and SN↑, TH in the ST and SN↑, Bax↓, Bcl-2↑, GFAP↓, Iba-1↓, NF-*κ*B↓, TNF-*α*↓, the Chao1 index↑, the number of observed OTUs↑, the Shannon index↑, Rikenellaceae↑, Vallitaleaceae↑, Alistipes↑, Vallitalea↑, Lachnoclostridium↑, Pseudoclostridium↑, Bacteroides xylanolyticus↑, Vallitalea pronyensis↑, Clostridium aerotolerans↑, Pseudoclostridium thermosuccinogenes↑, Roseburia faecis↑, holdemania↓, frisingicoccus↓, aestuariispira↓, sporobacter↓, rhodospirillum↓, Bifidobacterium↓, turicibacter↓, marvinbryantia↓, desulfovibrio↓, phascolarctobacterium↓, erysipelatoclostridium↓, butyricimonas↑, gracilibacter↑, phocea↑, desulfitobacterium↑, oscillibacter↑, acutalibacter↑, flintibacter↑, photosynthesis↑, glutathione metabolism↑, tetracycline biosynthesis↑, photosynthetic proteins↑, methane metabolism↓, drug metabolism↓

Park et al. (2003) [66]	6-OHDA rats	MA	Acu1: GB34, LR3; Acu2: LI4, LI11	Turned at a rate of two spins per second for 15 s, retained for 60 s	Rotational behavior test: the average net number of turns/min (Acu1)↓	TH-immunoreactive neurons in the ipsilateral SN (Acu1)↑, TH-immunoreactive terminals in the dorsolateral ST ipsilateral to the lesion (Acu1)↑, TrkB-positive cells in the SN (Acu1)↑

Lin et al. (2017) [67]	MPTP rats	EA	GB34, LR3	0/50 Hz, 1 mA for 20 minutes	Rotarod test: day 9 latency time↑; rotational behavior test: the average net number of turns/min↓	DA content in the ST↑, TH-positive neurons in the SN↑, degeneration in the ST↓, DA depletion in the ipsilateral side↓, mature BDNF, Bcl-2 and TH levels in the ipsilateral side↑, pAkt↑

Zhao et al. (2019) [68]	MFB rats	EA	GV20, GV29	2 Hz, 1.5 mA for 10 minutes	Rotarod test: latency time↑; tail suspension test: the immobility time↓	TH in the midbrain↑, BDNF^+^ cells↑, pAkt↑, pERK↑, functional TrkB FL↑, the ratio of TrkB FL/TrkB T1↑
	MFB rats	EA+K252a injection	GV20, GV29	2 Hz, 1.5 mA for 10 minutes	Rotarod test: latency time↓; tail suspension test: the immobility time↑	TH in the midbrain↓, BDNF^+^ cells↓, pAkt↓, pERK1/2↓

Kim et al. (2011) [69]	MPTP mice	MA	GB34	Turned at a rate of two spins per second for 15 s	Rotarod test: latency time↑, the ORP scores↑	pAkt↑
	MPTP mice	MA+LY294002	GB34	Turned at a rate of two spins per second for 15 s	Rotarod test: latency time↓, the ORP scores↓	pAkt↓, TH-positive dopaminergic neurons in SN↓, TH-positive dopaminergic fibers in ST↓

Hwang et al. (2019) [70]	MPTP mice	MA+Chunggan formula	GB34	Turned at a rate of two spins per second for 30s	Rotarod test: latency time↑; cylinder test: the number of wall touches↑; pole test: descending times↓	TH-positive dopaminergic neurons in the SNpc↑, TH protein in the SNpc and ST↑, pI*κ*B*α*↓, pAkt↑, pGSK3*β*↑, pERK↑, pCREB↑, BDNF↑

Jeon et al. (2017) [71]	MPTP mice	MA	Acu1: GB34; Acu2: HT7	Turned at a rate of two spins per second for 30 s	Pole test: day 5 descending times (GB34)↓, day 12 descending times↓	TH-positive cells in the SN (GB34)↑, TH immunoreactivity in the ST (GB34)↑, BrdU-positive cells in the SVZ (GB34)↑, BrdU/DCX-double stained cells in the SVZ↓, BrdU/GFAP-double stained cells in the SVZ (GB34)↑, GFAP-positive cells in the ST↑

Yu et al. (2010) [38]	6-OHDA rats	MA	GB34, LR3, ST36, SP10	Left in acupoints for 20 minutes	Rotational behavior test: the average net number of turns/min↓	SOD↑, GSH-Px↑, GSH↑, MDA↓

Wang et al. (2011) [47]	MPTP mice	EA	ST36, SP6	0/100 Hz, 1-1.25-1.5 mA for 30 minutes	—	TH immunoreactivity↑, day 3 DA, DOPAC, and HVA in the ST↑, H_2_O_2_ content in the ST↓, day 3 GSH content↑, days 3 and 7 GSH-Px↓, days 7 and 14 SOD activity↑, day 7 MDA content in the ST↓

Lee et al. (2018) [72]	MPTP mice	MA	Acu1: GB34; Acu2: SI3	Turned at a rate of two spins per second for 30 s	Pole test: descending times (GB34)↓	TH-positive neurons in the SN (GB34)↑, caspase-3 in the ST (GB34)↓, DJ-1 in the ST (GB34)↑, SOD activity (GB34)↑, CAT activity (GB34)↑

Kang et al. (2007) [73]	MPTP mice	MA	GB34, LR3	Turned at a rate of two spins per second for 15 s	—	TH-immunoreactive in the ST↑, TH-positive neurons in the SN↑, MAC-1↓, COX-2↓, iNOS↓, DA, DOPAC, and HVA in the ST↑

Li et al. (2019) [74]	6-OHDA rats	MA	CV12, ST25, CV4	Left in acupoints for 15 minutes	Rotational behavior test: the average net number of turns/min↓	TH-positive neurons in the SN↑, TNF-*α*↓, IL-1*β*↓, Fe levels↓, ratio of Fpn1/DMT1 mRNA↓, *α*-syn in the duodenum↓

Yang et al. (2017) [75]	MPTP mice	MA	GB34	Turned at 2 Hz for 15 s	—	NS

Kim et al. (2019) [76]	Pitx3-deficient ak/ak mice	MA+L-DOPA	GB34	Turned at a rate of two spins per second for 15 s	Three-paw dyskinesia test, abnormal three-paw movements↓; AIM scores↓	Pmch↑, Tac2 mRNA↑, Lcn2 mRNA↑
	Pitx3-deficient ak/ak mice	MA+L-DOPA+TC-MCH7c	GB34	Turned at a rate of two spins per second for 15 s	Three-paw dyskinesia test: abnormal three-paw movements↑	—
	6-OHDA mice	MA+L-DOPA	GB34	Turned at a rate of two spins per second for 15 s	AIM scores↓	Pmch↑

Wattanathorn and Sutalangka (2014) [77]	6-OHDA rats	Laser acupuncture	HT7	Continuous blue laser beam, wavelength of 405 nm, output power 100 mW, a spot diameter of 500 *μ*m for 10 minutes	Morris water maze: escape latency (days 7 and 14)↓, retention time (day 14)↑	Neuron density in CA3 and dentate gyrus↑, AChE in the hippocampus↓, MAO-B activity in the hippocampus↓, GSH-Px activity in the right SN↑, MDA in the right SN↓

Park et al. (2017) [78]	MPTP mice	MA	GB34	Turned at a rate of two spins per second for 15 s	Rotarod test: latency time↑; cylinder test: the number of wall touches↑	TH-positive neurons in the SN↑, dopaminergic fibers in the ST↑, Pmch in the hypothalamus↑, double-labeled c-Fos^+^/MCH^+^ cells↑, MCH concentration in the SN↑, the expression levels of TH and synaptophysin in the SN↑, pAkt↑, and pCREB↑
	MPTP mice	MA+TC-MCH7c	GB34	Turned at a rate of two spins per second for 15 s	Rotarod test: latency time↓; cylinder test: the number of wall touches↓	TH-positive neurons in the SN↓, dopaminergic fibers in the ST↓
	Pitx3^−/+^ mice	MA	GB34	Turned at a rate of two spins per second for 15 s	Rotarod test: latency time↑	—
	Pitx3^−/+^ mice	MA+TC-MCH7c	GB34	Turned at a rate of two spins per second for 15 s	Rotarod test: latency time↓	—
	A53T Tg mice	MA	GB34	Turned at a rate of two spins per second for 15 s	Rotarod test: latency time↑; cylinder test: the number of wall touches↑	pGSK-3*β* and pAMPK in the SN↑, BDNF expression in the SN↑, synuclein expression in the SN↓

MPTP: 1-methyl-4-phenyl-1,2,3,6-tetrahydropyridine; MA: manual acupuncture; GB34: *Yanglingquan*; Grm5: glutamate receptor metabotropic 5; Itpr1: inositol 1,4,5-triphosphate receptor 1; Itpr2: inositol 1,4,5-triphosphate receptor 2; Pde1c: phospho-diesterase 1C; Prkcb1: protein kinase beta 1; Plcb4: phospholipase C beta 4; Atp2b3: ATPase calcium-transporting plasma membrane 3; Slc8a3: solute carrier family 8, sodium/calcium exchange member 3; Htr5a: 5-hydroxytryptamine (serotonin) receptor 5A; Adcy1: adenylate cyclase 1; Drd5: dopamine receptor 5; Npy1r: neuropeptide Y receptor Y1; Gpr156: G protein-coupled receptor 156; Cxcl10: chemokine (C–X–C motif) ligand 10; Rxfp2: relaxin/insulin-like family peptide receptor 2; Agtrl1: angiotensin receptor-like 1; Inhbb: inhibin beta-B; Cxcl5: chemokine (C–X–C motif) ligand 5; IL21: interleukin 21; Cox6a1: cytochrome c oxidase, subunit VI a, polypeptide 1; Uqcrq: ubiquinol-cytochrome c reductase, complex III subunit VII; Ndufc1: NADH dehydrogenase (ubiquinone) 1, subcomplex unknown, 1; Atp5e: ATP synthase, epsilon subunit; Mapt: microtubule-associated protein tau; Ngfr: nerve growth factor receptor (TNFR superfamily, member 16); Nefh: neurofilament, heavy polypeptide; LR3: *Taichong*; TH: tyrosine hydroxylase; ST: striatum; SNpc: substantia nigra pars compacta; DAT: dopamine transporter; Ctla2a: cytotoxic T lymphocyte-associated protein 2 alpha; EG383229: predicted gene EG383229; Ppbp: proplatelet basic protein; Ube2l6: ubiquitin-conjugating enzyme E2L 6; EG665033: predicted gene EG665033; ENSMUSG00000055323: predicted gene ENSMUSG00000055323; Obox6: oocyte-specific homeobox 6; Pbp2: phosphatidylethanolamine-binding protein 2; Tmem150: transmembrane protein 150; Dusp4: dual specificity phosphatase 4; Mafg: v-maf musculoaponeurotic fibrosarcoma oncogene family, protein G; Pgm5: phosphoglucomutase 5; Ndph: Norrie disease homolog; Dnase1l2: deoxyribonuclease 1-like 2; Ucp2: uncoupling protein 2; Sp2: Sp2 transcription factor; Serinc2: serine incorporator 2; Cdh1: cadherin 1; Slc6a13: solute carrier family 6 (neurotransmitter transporter, GABA), member 13; Tph2: tryptophan hydroxylase 2; Mpzl2: myelin protein zero-like 2; Serping1: the serine (or cysteine) peptidase inhibitor, clade G, member 1; Itih2: interalpha trypsin inhibitor, heavy chain 2; Slc6a20a: SLC6 (neurotransmitter transporter), member 20A; Slc6a4: SLC6 (neurotransmitter transporter, serotonin), member 4; Ucma: upper zone of growth plate and cartilage matrix associated; Rdh9: retinol dehydrogenase 9; 4921530L21Rik: RIKEN cDNA 4921530L21 gene; Gm13931: predicted gene 13931; EA: electroacupuncture; CyPA: cyclophilin A; MBP: myelin basic protein; Ube2N: ubiquitin-conjugating enzyme E2N; LMP6: proteasome subunit C7-I; LMP2: 20S proteasome subunit; eIF5A: eukaryotic translation initiation factor 5A; CLP: coactosin-like protein; NSF: N-ethylmaleimidesensitive fusion protein; ST36: *Zusanli*; SP6: *Sanyinjiao*; 6-OHDA: 6-hydroxydopamine; LI4: *Hegu*; RA: retained acupuncture; PC7: *Daling*; DA: dopamine; [^123^I] IBZM: [^123^I] iodobenzamide; HVA: homovanillic acid; *α*-syn: *α*-synuclein; SGK1: serum/glucocorticoid-regulated kinase 1; p-*α*-syn: phosphorylated *α*-synuclein; ORP: overall rod performance; LC3-II: microtubule-associated protein 1 light chain 3 II; LAMP1: lysosome-associated membrane protein 1; CV4: *Guanyuan*; GV16: *Fengfu*; p-mTOR: phosphorylated mammalian target of rapamycin; p-p70S6k: phosphorylated ribosomal protein S6 kinase; BVA: bee venom acupuncture; Bax: Bcl-2-associated X protein; MDA: malondialdehyde; GSH: glutathione; TNF-*α*: tumor necrosis factor-*α*; IL-1*β*: interleukin-1*β*; Bcl-2: B cell lymphoma-2; GFAP: glial fibrillary acidic protein; Iba-1: ionized calcium-binding adaptor molecule-1; NF-*κ*B: nuclear factor kappa-B; OTUs: operational taxonomic units; COX-2: cyclooxygenase; iNOS: inducible NO synthase; p-c-Jun: phosphorylated c-Jun kinase; MFB: medial forebrain bundle; GV14: *Dazhui*; GV20: *Baihui*; BDNF: brain-derived neurotrophic factor; VTA: ventral tegmental area; GDNF: glial cell-derived neurotrophic factor; SNpr: substantia nigra pars reticulata; GP: globus pallidus; LI11: *Quchi*; TrkB: tyrosine kinase receptor B; GV29: *Yintang*; PKB/Akt: protein kinase B; p-Akt: phosphorylated Akt; pERK: phosphorylated extracellular-regulated protein kinase; TrkB FL: full-length TrkB; TrkB T1: splicing truncated isoforms TrkB; pI*κ*B*α*: phosphorylated inhibitor kappa B alpha; pGSK3*β*: phosphorylated glycogen synthase kinase 3*β*; pCREB: phosphorylated cAMP-response element-binding protein; HT7: *Shenmen*; BrdU: 5-bromo-2′-deoxyuridine; SVZ: subventricular zone; DCX: doublecortin; SP10: *Xuehai*; SOD: superoxide dismutase; GSH-Px: glutathione peroxidase; DOPAC: dihydroxyphenylacetic acid; SI3: *Houxi*; CAT: catalase; MAC-1: macrophage antigen complex-1; CV12: *Zhongwan*; ST25: *Tianshu*; Fpn1: ferroportin1; DMT1: recombinant divalent metal transporter 1; NS: no significance; L-DOPA: levodopa; AIM: abnormal involuntary movement; Pmch: pro-melanin-concentrating hormone; Tac2: tachykinin 2; Lcn2: lipocalin 2; CA3: the CA3 region of the hippocampus; AChE: acetyl cholinesterase; MAO-B: monoamine oxidase-B; MCH: melanin-concentrating hormone; pAMPK: phosphorylated cAMP-response phosphorylated adenosine 5-monophosphate-activated protein kinase.

**Table 3:** The neural circuit mechanisms of acupuncture for Parkinson's disease in clinical researches.

Study	Clinical trial design	Clinical condition	Intervention	Comparison	Acupoints	Acupuncture parameters	Outcomes
Chae et al. (2009) [98]	Controlled trial	PD patients (mild)	Verum acupuncture (*n* = 10)	Covert placebo/overt placebo (*n* = 10)	Left GB34	10 mm, manually rotated clockwise and counterclockwise once per second (1 Hz), 60 seconds, then withdrawn for 120 seconds, only once	The putamen and the primary motor cortex↑, motor function↑ (VA vs. CP)

Yeo et al. (2012) [99]	RCT	Idiopathic PD patients	Acupuncture (*n* = 12)	Acupuncture on healthy participants (*n* = 12)	Right GB34	1 cm, remained in the skin for 1 min and rotated bidirectionally for 1 min, needle remained in the skin without rotation for 1 min and then the pattern of 1 min rotation and 1 min rest was repeated, only once	Neural responses in the SN, caudate, thalamus, and putamen (impaired caused by PD)↑

Yeo et al. (2014) [100]	RCT	Idiopathic PD patients	Acupuncture (*n* = 12)	Acupuncture on healthy participants (*n* = 12)	Right GB34	1 cm, for “stimulated” blocks: rotated bidirectionally at 1 Hz; for “not stimulated” blocks: the needle was only inserted into the skin and then left in place, only once	Neural responses in the prefrontal cortex, precentral gyrus, and putamen in PD patients↑

Li et al. (2018) [101]	Controlled trial	PD patients with tremor	Acupuncture+L-DOPA (*n* = 14)	Sham acupuncture+L-DOPA (*n* = 14)	DU20, bilateral GB20, and the chorea-tremor controlled zone	2-3 cm, reinforcing-reducing method, twirling every 10 minutes, 30 minutes, twice weekly, 12 weeks	Clinical evaluation: UPDRS (II and III) scores↓, neural responses (SN, caudate, thalamus, and putamen)↑

Huang et al. (2010) [102]	RCT	PD patients	Acupuncture+L-DOPA (*n* = 5)	L-DOPA (*n* = 5)	Bilateral MS6, MS4, MS8, MS9, MS14	Continuous wave, 50 Hz, 2-4 mA, 30 minutes, six times a week, 5 weeks	rCBF (the frontal lobe, the occipital lobe, the basal ganglion, and the cerebellum in the most affected hemisphere)↑

Huang et al. (2009) [103]	RCT	PD patients	Acupuncture+Madopar (*n* = 5)	Madopar (*n* = 5)	Bilateral MS6, MS4, MS8, MS9, MS14	Continuous wave, 50 Hz, 2–4 mA, 30 minutes, six times a week, 5 weeks	The glucose metabolisms (parietal, temporal, occipital lobes, the thalamus, and the cerebellum in the light-diseased hemisphere, and in parietal and occipital lobes of the severe-diseased hemisphere)↑

PD: Parkinson's disease; GB34: *Yanglingquan*; VA: verum acupuncture; CP: covert placebo; RCT: randomized controlled trial; SN: substantia nigra; L-DOPA: levodopa; DU20: *Baihui*; GB20: *Fengchi*; UPDRS: Unified Parkinson's Disease Rating Scale; MS6: the anterior oblique line of the vertex to temple; MS4: the lateral line III on forehead; MS8: the lateral line I of the vertex; MS9: the lateral line II of the vertex; MS14: the lower-lateral line of the occipital scalp; rCBF: regional cerebral blood flow.

**Table 4:** The neural circuit mechanisms of acupuncture for Parkinson's disease in basic researches.

Study	PD model	Intervention	Acupoints	Acupuncture parameters	Behavioral measurements	Biochemical measurements
Zhang et al. (2018) [104]	MPTP rhesus monkeys	EA	ST36, LI4	100 Hz, 3-4-5 mA for 30 minutes	The speed of movement↑, longer performance time of the affected hand↑	The BOLD activations of the caudate nucleus, putamen, M1, cingulate gyrus, and GPe↓

Lee et al. (2013) [97]	MPTP beagle dogs	RA	ST36	Rotated clockwise and counterclockwise to generate *Deqi*	—	The BOLD signal intensity in the ipsilateral parietal lobe, contralateral temporal lobe, basal ganglia, pons, and cerebellum↓

Khalil et al. (2015) [84]	Rotenone rats	BVA	GB34	—	Suspension test: latency time↑; cylinder test: the number of wall touches↑	Caspase-3 activity↓, caspase-3 genes↓, Bax genes↓, DNA damage↓, MDA↓, PON1 activity↑, GSH↑, TNF-*α*↓, IL-1*β*↓, DA↑, 5-HT↑, noradrenaline↑

Kim et al. (2011) [36]	MPTP mice	MA	GB34	Turned at a rate of two spins per second for 15 s	Rotarod test: the ORP scores↑	TH-positive neurons in the SN↑, Nissl-positive cells in the SNpc↑, dopaminergic fibers in the ST↑, HVA in the ST↑, dopamine turnover ratios (DOPAC/DA, HVA/DA, and DOPAC+HVA/DA)↑, dopamine efflux in the ST↑, pDARPP-32 at Thr34↓, FosB in the ST↓

Rui et al. (2013) [105]	6-OHDA rats	EA	SP6, GB34, ST36	0/2/100 Hz, 1-3 mA for 30 minutes	Rotational behavior test: the average net number of turns/min (100 Hz)↓	TH-positive neurons on the lesion side (100 Hz)↑, TH-positive dendritic fibers in the ST↑, DAT in the ST and SN (100 Hz)↑, D1R mRNA and protein in the ST (100 Hz)↑, D2R protein (100 Hz) in the ST↓

Jia et al. (2009) [106]	MFB rats	EA	GV14, GV20	0/2/100 Hz, 1-2-3 mA for 30 minutes	Rotational behavior test: the average net number of turns/min (100 Hz)↓	TH-positive neurons in the SN (100 Hz)↑, TH-positive dendritic fibers in the ipsilateral SNpr (100 Hz)↑, substance P content in the SN (100 Hz)↑, GAD67 mRNA↓

Jia et al. (2010) [107]	MFB rats	EA	GV14, GV20	0/2/100 Hz, 1-2-3 mA for 30 minutes	Rotarod test: week 2 treadmill occupancy time (100 Hz)↑	GABA levels in midbrain (100 Hz)↓

Kim et al. (2014) [108]	6-OHDA mice	MA+L-DOPA	GB34	Turned at a rate of two spins per second for 15 s	Cylinder test: the number of wall touches↑; AIM scores↓	FosB in the ST↓, GABA contents in the SNpr↓, Glu levels in the SN↓

Sun et al. (2012) [109]	MFB rats	EA	GV14, GV20	100 Hz, 1-2-3 mA for 30 minutes	Rotarod test: treadmill occupancy time↑; rotational behavior test: the average net number of turns/min↓	TH-positive neurons↑, ACh, Glu in the ST↓

PD: Parkinson's disease; MPTP: 1-methyl-4-phenyl-1,2,3,6-tetrahydropyridine; EA: electroacupuncture; ST36: *Zusanli*; LI4: *Hegu*; M1: primary motor cortex; GPe: globus pallidus externa; RA: retained acupuncture; BVA: bee venom acupuncture; GB34: *Yanglingquan*; Bax: Bcl-2-associated X protein; MDA: malondialdehyde; PON1: paraoxonase-1; GSH: glutathione; TNF-*α*: tumor necrosis factor-*α*; IL-1*β*: interleukin-1*β*; DA: dopamine; 5-HT: serotonin; MA: manual acupuncture; ORP: overall rod performance; SNpc: substantia nigra pars compacta; ST: striatum; HVA: homovanillic acid; DOPAC: dihydroxyphenylacetic acid; pDARPP-32: phosphorylated dopamine and adenosine 35-monophosphate-regulated phospho-protein; SP6: *Sanyinjiao*; DAT: dopamine transporter dopamine; D1R: D1 receptor; D2R: dopamine D2 receptor; MFB: medial forebrain bundle; GV14: Dazhui; GV20: Baihui; GAD67: glutamate decarboxylase-67; GABA: *γ*-aminobutyric acid; ACh: acetylcholine; Glu: glutamate.

## Abbreviations

Ach: Acetylcholine
AChE: Acetylcholinesterase
*α*-syn: *α*-Synuclein
Bcl-2: B cell lymphoma-2
BDNF: Brain-derived neurotrophic factor
BrdU: 5-Bromo-2′-deoxyuridine
BVA: Bee venom acupuncture
Caspase: Cysteinyl aspartate-specific proteinase
CAT: Catalase
COX-2: Cyclooxygenase-2
CTC: Cerebello-thalamo-cortical
DA: Dopamine
DAT: Dopamine transporter
D1R: Dopamine D1 receptor
D2R: Dopamine D2 receptor
EA: Electroacupuncture
EBM: Evidence-based medicine
4E-BP1: 4E-binding protein 1
fMRI: Functional magnetic resonance imaging
GABA: *γ*-Aminobutyric acid
GDNF: Glial cell-derived neurotrophic factor
Glu: Glutamate
GSH: Glutathione
GSH-Px: Glutathione peroxidase
5-HT: Serotonin
IL-1*β*: Interleukin-1*β*
iNOS: Inducible nitric oxide synthase
JNK: c-Jun N-terminal kinase
LAMP1: Lysosomal-associated membrane protein 1
LAS: Lysosomal autophagy system
LC3-II: Microtubule-associated protein 1 light chain 3 II
L-DOPA: Levodopa
LID: L-DOPA-induced dyskinesia
MA: Manual acupuncture
MAO-B: Monoamine oxidase B
MAs: Meta-analyses
MCH: Melanin concentration hormone
MDA: Malondialdehyde
MFB: Medial forebrain bundle
mTOR: Mammalian target of rapamycin
MPTP: 1-Methyl-4-phenyl-1,2,3,6-tetrahydropyridine
NE: Norepinephrine
NTFs: Neurotrophic factors
6-OHDA: 6-Hydroxydopamine
PD: Parkinson's disease
PET: Positron emission tomography
rCBF: Regional cerebral blood flow
RCTs: Randomized controlled trials
ROS: Reactive oxygen species
SGK1: Serum/glucocorticoid-regulated kinase 1
SN: Substantia nigra
SNpc: Substantia nigra pars compacta
SOD: Superoxide dismutase
SPECT: Single-photon emission computed tomography
SRs: Systematic reviews
ST: Striatum
TNF-*α*: Tumor necrosis factor-*α*
TrkB: Tyrosine kinase receptor B
UPDRS: Unified Parkinson's Disease Rating Scale.
